# A Rare Case of Lemierre’s Syndrome due to Veillonella Parvula: A Dangerous and Forgotten Complication of a Septic Condition

**DOI:** 10.1007/s12070-024-04615-w

**Published:** 2024-04-02

**Authors:** Manuela Montatore, Antonio Zagaria, Federica Masino, Giacomo Fascia, Michele Debitonto, Giuseppe Guglielmi

**Affiliations:** 1 Department of Clinical and Experimental Medicine, Foggia University School of Medicine, Viale L. Pinto 1, Foggia, Foggia, FG 71121 Italy; 2 Department of Intensive Care and Anaesthesiology, “Dimiccoli” Hospital, Viale Ippocrate 15, Barletta, BT 70051 Italy; 3 Radiology Unit, ‘‘Dimiccoli’’ Hospital, Viale Ippocrate, 15, Barletta (BT), BT 70051 Italy; 4 Radiology Unit, “IRCCS Casa Sollievo della Sofferenza” Hospital, Viale Cappuccini 1, San Giovanni Rotondo, FG 71013 Italy

**Keywords:** Leimerre’s syndrome, Septic emboli, Oropharyngeal infection, thrombophlebitis, Vein thrombosis, Veillonella parvula, Enterobacter cloacae

## Abstract

This clinical case presents an unusual case of Lemierre’s syndrome (LS) in a young woman of 38-year-old. She arrived in the Emergency Department with a high fever and pharyngology resistant to antibiotic therapy with clarithromycin, ceftriaxone, and cortisone for two weeks. At the blood sampling, there is a marked leucocytosis, and the advice of the otolaryngologist is required given the strong pain in the throat. Due to the tonsillar abscess, a neck CT with a contrast medium is necessary for the otolaryngologist’s opinion. The CT shows thrombosis of the jugular vein and left subclavian, with thickening of soft perivascular tissues; these findings suggest Lemierre’s syndrome: a septic thrombophlebitis of the jugular vein that occurs as a complication of a peritonsillar abscess. The diagnostic process is then completed with a chest HR-CT, which reveals lung density and excavation areas suggesting tuberculosis. Blood culture reveals the presence of Veillonella Parvula (an anaerobic gram-negative coccus), sputum culture reveals the presence of some colonies of Enterobacter cloacae complex, real-time PCR examination on sputum reveals the presence of Streptococcus Pneumoniae and the borderline presence of rhinovirus. Microbiologists, after these results and neck and chest CT with a contrast agent, agree with the diagnosis of suspected LS at an early stage: a septic dissemination fortunately limited only to the neck and lungs region.

## Introduction

Lemierre’s syndrome (LS) is a potentially fatal pharyngitis sequela; an oropharyngeal infection causes subsequent septic thrombophlebitis of the internal jugular vein, with embolization of the lungs and other organs. This diagnosis requires a high index of suspicion in the workup of pharyngitis and should be treated aggressively just once.

Anaerobic Gram-negative bacteria are often involved, especially by Fusobacterium Necrophorum, rarely due to Veillonella Parvula an anaerobic Gram-negative coccus found in the typical human flora.

It has rarely been discovered as a pathogen in humans, and the most common illness caused by V. Parvula is mostly osteomyelitis [1].

Although Lemierre’s Syndrome is uncommon and very dangerous, the physician should be aware of it since patients with LS require immediate and effective antibiotic therapy to avoid severe complications. In fact, with the proper antibiotic regimen, complete healing is possible.

## Case Presentation

A 38-year-old woman arrived at the Emergency Department with a sore throat and fever (about 38 degrees) for about two weeks, resistant to antibiotic therapy ((with ceftriaxone, clarithromycin for 6 days, and 3 days of corticosteroids), the suspicion of a tonsil abscess is raised, confirmed by the otolaryngologist (ENT).

**Fig. 1:**
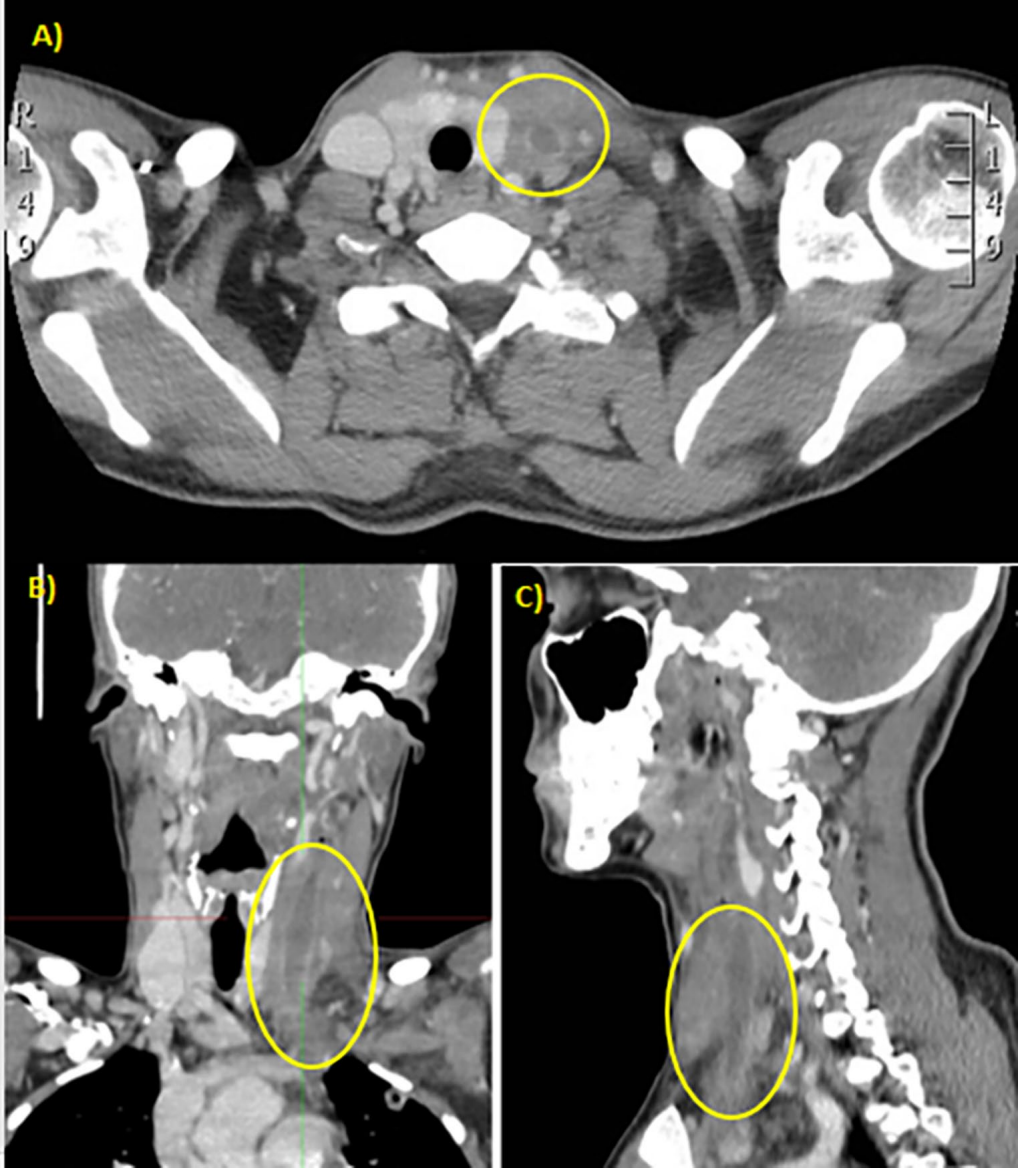
A CT of the neck, with contrast medium, shows that there is thrombosis of the jugular vein and subclavian left with thickening of soft perivascular tissues **(A)** in the axial section; **(B)** in the coronal section; **(C)** in the sagittal section

The patient is asthenic and has a feverish state, pharyngitis, and pale skin. There is marked neutrophil leukocytosis (51,000 WBCs per microliter), CRP is 7.2 mg/dl, PCT 29.5 ng/dl, and COVID buffer is negative. After the state of pregnancy was excluded, the ENT consulting asked for a neck CT with a contrast medium (Fig. 1).

**Fig. 2:**
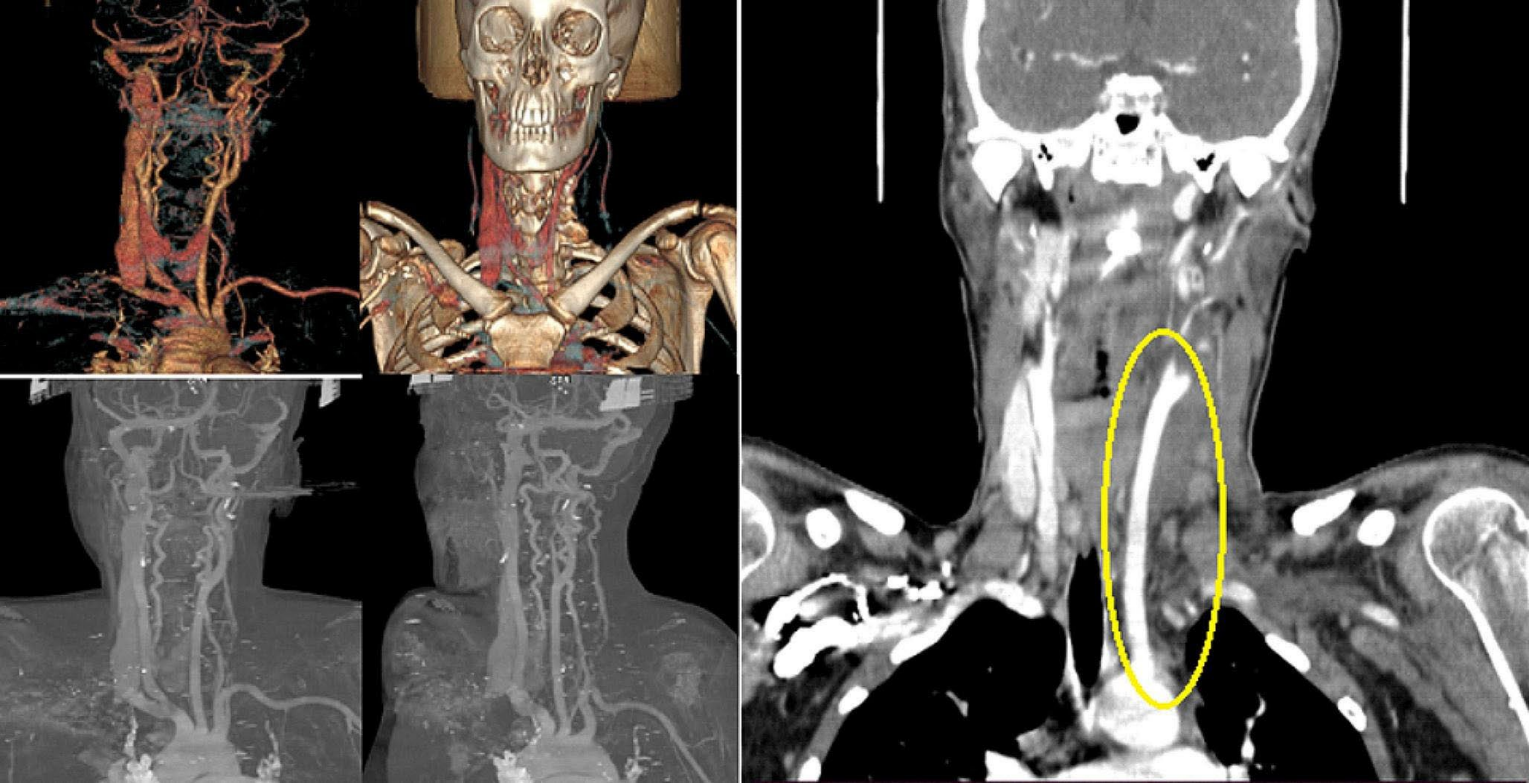
Angiotomography of the neck vessels. A filling defect is observed along the entire length of the left internal jugular vein

CT shows a complete opacification defect, thrombotic with some endoluminal air bubbles (suspected in the beginning for a bacterial infection and anaerobic metabolism), of the left jugular vein and some of its tributary branches, associated with thickening of perivascular soft tissue (Fig. 2).

The few scans through the pulmonary apices show multiple thickening pseudo-nodular areas, some excavated, that require a deepening with CT of the chest, to be distinct from further suspicion of tuberculosis; in fact, rarely also Mycobacterium tuberculosis can cause an LS, alone or in coinfection with Levinea sp [2].

**Fig. 3:**
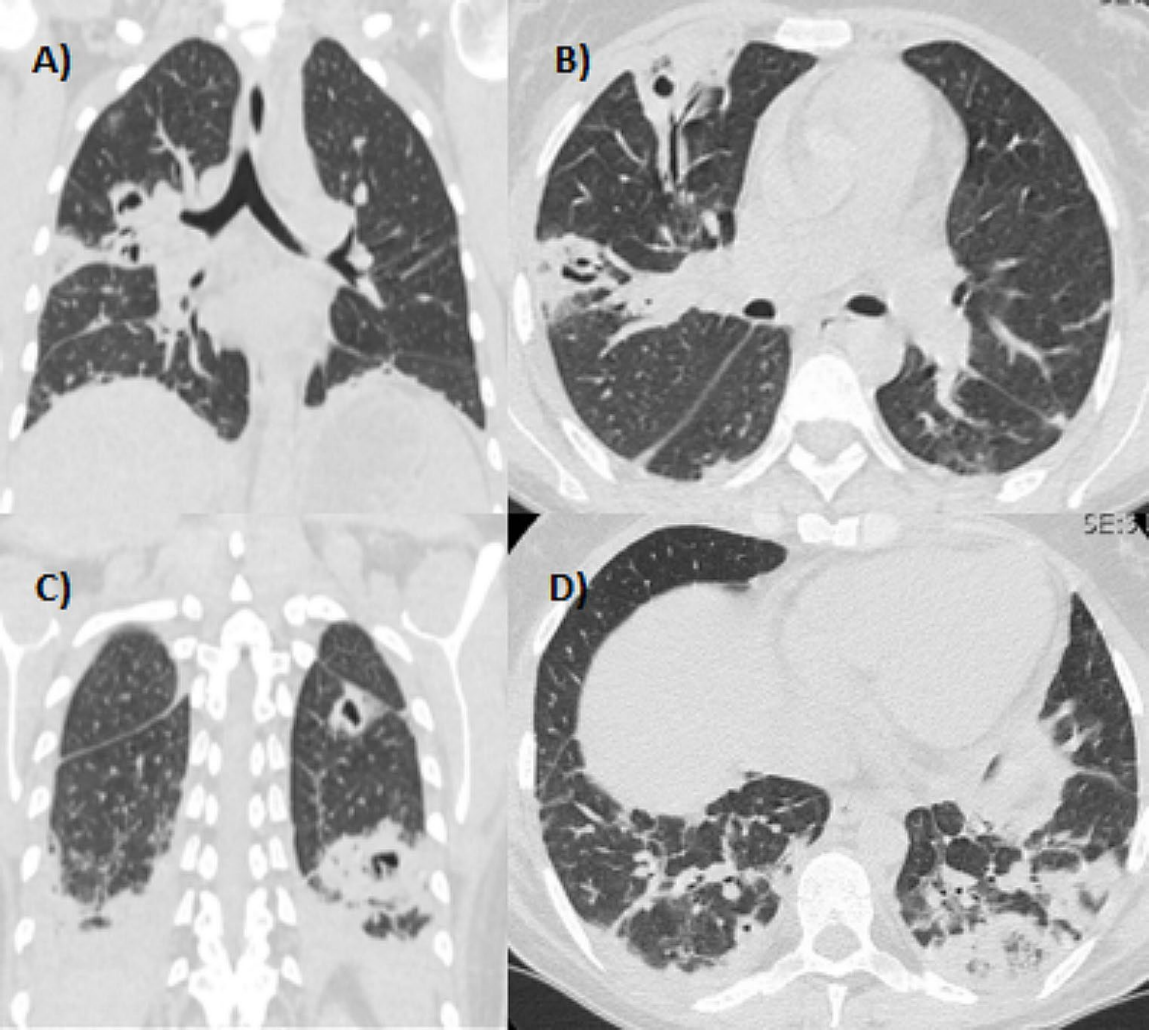
The HR-CT of the chest reveals pulmonary densities and areas of excavations at the posterior-basal segment of the LIS and anteroposterior of LSD probable infectious hypothesis, also caused by Mycobacterium tuberculosis. **A**) and **B**), a coronal section and an axial section, show the condition on the apex of the lungs. **C**) and **D**), a coronal section and an axial section, show the condition of the basal parenchyma of the lungs

A high-resolution CT scan of the chest is then performed on suspicion of a septic infection starting from the lungs: there is a pulmonary thickening with contextual excavation areas [3, 4] (Fig. 3).

The Department of Anaesthesia and Resuscitation evaluates the patient in isolation for infectious risk. It is vigilant and collaborative, perfectly oriented in time and space (GCS: 15), without a deficit of the state of consciousness, and isochoric, iso-cyclic, and photoreactive pupils; she moves her four limbs and does not present any deficit of strength or side signs.

The patient is in spontaneous respiration at 4 L per minute, with TC 38 fever, blood pressure 110/70 mmHg, and heart rate 80 bpm.

The Resuscitation Department, after doing a blood-gas analysis (P02 80, PC02 30, ph. 7.5, LAC negative), puts the increasing suspicion of Lemierre’s Syndrome and requires an imaging overview of the missing segments, so a skull CT and a US of the abdomen that are both negative to exclude the dissemination of the septic emboli, in a picture of sepsis with pulmonary excavations.

Finally, the Department of Infectious Diseases recommends research of Mycobacterium Tuberculosis (PCR, microscopic, and cultural examination) on 3 samples of sputum or BAL and Antigen-serology research for Candida and Aspergillus and Galactomannan on expectorate (or on BAL). QuantiFERON and HIV tests are also performed.

The physician recommends performing at least two blood cultures and culture tests on sputum, serology for atypical bacteria, urinary antigens for legionella and pneumococcus, and NPS (nasopharyngeal swab) for the virus and respiratory bacteria research. They must also perform urinalysis and urine collection in addition.

After taking the various samples requested by the Department of Infectious Diseases, antibiotic therapy with piperacillin/tazobactam 4.5 gr for 5 days 4 times a day, in 100 cc of physiological solution (for an infusion duration of at least 3 h) and the light a value of procalcitonin to 25 ng /ml is also added vancomycin for 1 gr day, 2 times a day in 250 cc of saline (90 min of infusion); the patient is placed in preventive respiratory isolation.

Following an evaluation at the Department of Cardiology: asymptomatic patient for typical Angor, dyspnoea, and eupnoea. Absence of clinical, haemato-chemical, and instrumental signs of cardiological acuity in place. The patient will be admitted to the Infectious Disease ward until the clinical picture has been addressed [5, 6].

After a few days comes the result of microbiological evaluations: the sputum is positive for Enterobacter cloacae complex, real-time positive PCR weak for rhinovirus and positive for streptococcus pneumoniae, positive for culture on blood for Parvula Veillonella.

**Fig. 4:**
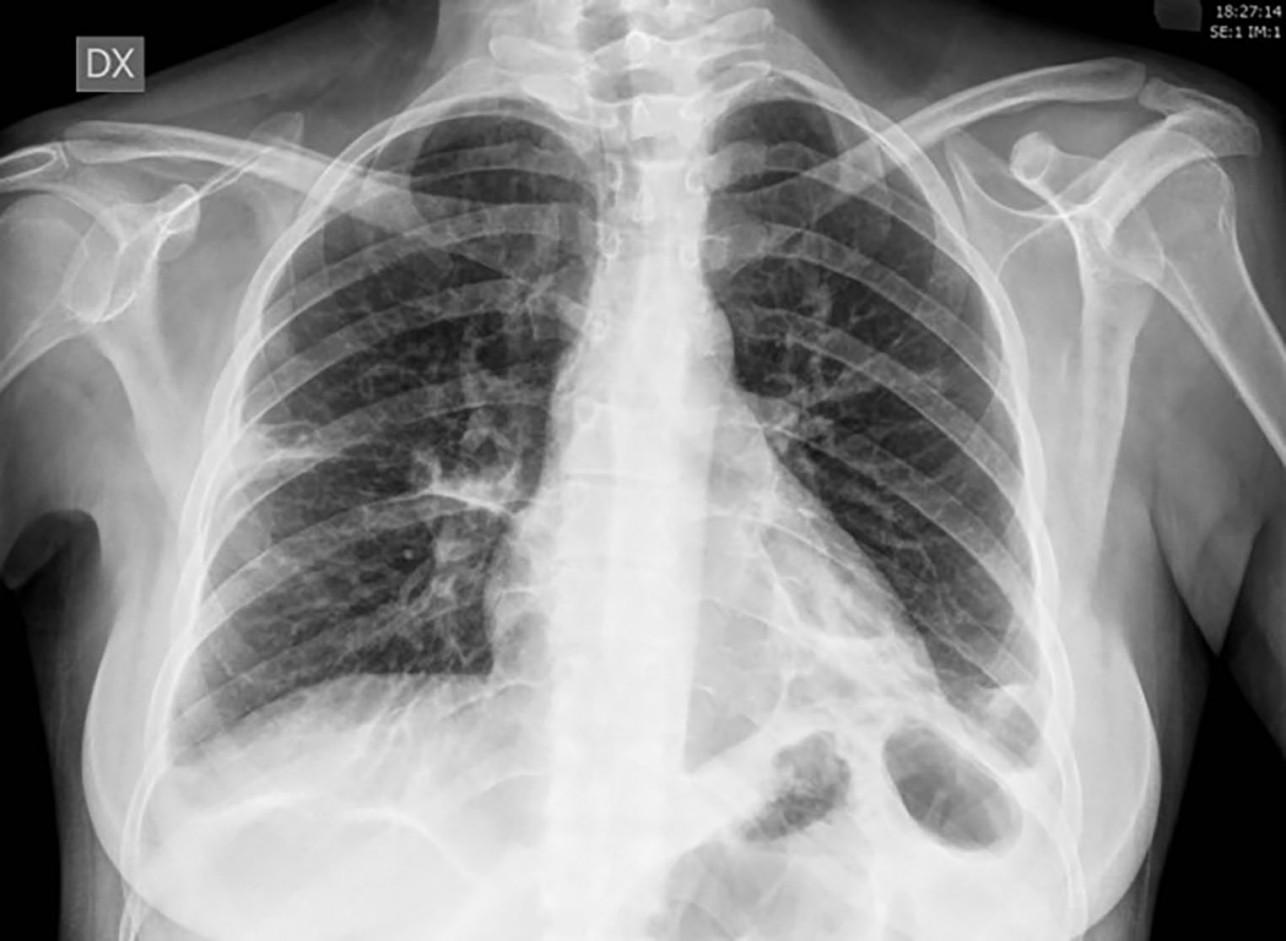
The X-ray examination was performed in a single projection and showed a circumscribed parenchymal thickening at the level of the basal pulmonary field on the left. There is a dystelectasis stria at the peri-scissure site on the right, with traction relative to the pleura. Moderately thickened the peri-bronco vascular interstitial

A diagnosis of Lemierre’s syndrome for sepsis by Veillonella Parvula was made. To assess the response to antibiotic therapy and progress, a chest X-ray is required: showing a slow resolution of the infectious lung condition (Fig. 4). Only when the patient’s clinical conditions improved, she was discharged, but she remained under strict follow-up: an echography of the neck vessels was programmed for the next three months.

## Discussion

The presence of septic pulmonary thromboembolism, as well as thrombosis of the left jugular vein in a patient who had previously had an upper respiratory tract infection, leads us to postulate Lemierre’s syndrome.

André Lemierre’s first characterized illness in 1936, and it has been widely and indiscriminately applied to clinical cases that involve any of the following: acute pharyngitis with septic thrombophlebitis, distant septic embolism, or culture positive for Gram-negative bacterial especially fusobacterium.

Lemierre’s syndrome, sometimes known as ‘necrobacillosis,’ is a type of post-anginal characterized by acute oropharyngeal infection and secondary thrombophlebitis of the internal jugular vein.

Septic emboli are commonly found in the lungs; however, they can also harm the digestive organs. Fusobacterium Necrophorum a strictly anaerobic gram-negative bacteria, causes this condition, which is sometimes associated with other diseases [7].

Very rarely LS is caused by Veillonella Parvula: a type of bacteria habiting the oral cavity and gastrointestinal tract in humans and rarely causing severe infections, such as bacteraemia, meningitis, endocarditis, prosthetic joint infection, pulmonary infection, and vertebral osteomyelitis [1, 8].

The patient usually comes with a high temperature, neck discomfort, malaise, and dyspnoea one week following the onset of angina.

The lungs are the most prevalent site of metastatic infection; thus, imaging should include a chest radiograph to look for septic emboli and other pulmonary problems. Bacterial metastasis can also cause septic arthritis, osteomyelitis, meningitis, pericarditis, and hepatic abscesses [9–11].

Ultrasound, CT of the neck with contrast, and in some cases magnetic resonance imaging (MRI) are other imaging modalities used to evaluate for septic thrombosis of the internal jugular vein [4].

Currently, there are no specific care guidelines for LS. However, the mainstay of treatment for Lemierre’s syndrome is antibiotic therapy such as a mix of macrolides, second or third-generation cephalosporins, and anti-coagulants are commonly used [12].

While surgical drainage can effectively eliminate septic foci, it comes with significant difficulties and side consequences: for example, in cases of abscess formation, respiratory discomfort due to pulmonary thrombosis, metastasis, and thrombus extension into the mediastinum or cerebrum. To control the infection, surgical incision and draining of the abscess at the afflicted locations may be required.

Anticoagulation therapy in Lemierre’s syndrome is still debatable: however, anticoagulation is frequently suggested when the thrombus spreads into the cerebral sinuses.

## Conclusion

Whenever we are faced with banal pharyngotonsillitis, we must try not to underestimate the symptoms especially if the local infection is associated with a septic picture, the suspicion of Lemierre’s Syndrome arises. First, it is necessary to search the ultrasound for a possible thrombosis of the internal jugular vein and deepen the diagnostic framework in search of any systemic septic emboli. Only with a rapid diagnosis, a precise imaging path, and setting a useful and timely antibiotic therapy can serious situations be avoided, even the exitus.
